# Understanding the factors that affect households’ investment decisions required by the energy transition

**DOI:** 10.1371/journal.pone.0297222

**Published:** 2024-03-21

**Authors:** Armando Aguayo-Mendoza, Ane Irizar-Arrieta, Diego Casado-Mansilla, Cruz E. Borges

**Affiliations:** 1 DeustoTech, Faculty of Engineering, University of Deusto, Bilbao, Spain; 2 Faculty of New Interactive Technologies, Universidad EUNEIZ, Vitoria, Spain; National Technical University of Athens: Ethniko Metsobio Polytechneio, GREECE

## Abstract

In energy systems’ economic models, people’s behaviour is often underestimated, and they are generally unaware of how habits impact energy efficiency. Improving efficiency is challenging, and recommendations alone may not be sufficient. Changing behaviour requires understanding the direct impact of needs and habits on energy efficiency. This research introduces a methodology that retrieves human expert knowledge from four key aspects of the current energy transition: everyday appliances, buildings, mobility, flexibility, and energy efficiency. The aim is to examine the causal relationship between energy consumption and human behaviour, gaining a deeper understanding of the links among the factors that drive final energy consumers to change habits through the adoption of energy-saving measures. Working in collaboration with expert panels, this study provides a methodology for extracting expert human knowledge based on a set of future energy transition scenarios aligned with the achievement of the Paris Agreement, a taxonomy of 32 factors that have a strong influence on households’ investment decisions, and the results of a survey that characterises the European population through the 32-factor taxonomy and some socioeconomic conditions. In addition, the survey included a sample of the Latin American population to analyse how socioeconomic conditions (region, education, gender, etc.) influence the prioritisation of these factors. We discuss the high priority given to competence and autonomy over financial factors by inhabitants of the European Union residential sector. We provide an analysis of the factors through which other similar projects are focused and on which we converge. In addition, we contribute by presenting the hierarchy of priorities assigned by people. This highlights the importance for policymakers to take these aspects seriously when implementing energy policy interventions that go beyond purely financial measures and fiscal incentives.

## Introduction

The techno-economic model of the current energy system—which emphasises technology and cost-cutting, and where human behaviour is viewed as a minor consideration—has dominated discussions and policy agendas related to encouraging energy efficiency [1] initiatives in households within the European Union (EU) for the past few decades. This strategy places a lot of emphasis on the adoption of new technologies, regularly reviewing the cost savings, and paying attention to return on investment (ROI). The energy transition away from a social commitment can lead to economic and demographic contractions in the affected areas. This, in turn, can result in energy poverty due to a loss of income and its impact on the quality of life [2]. The energy transition may also impact vulnerable households, bringing to light additional dimensions of energy poverty, such as transport poverty [3]. The importance of human behaviour has frequently been seen in this approach as a minor or even unimportant consideration. Behavioural economics’ introduction, however, [4], has started to make it possible to incorporate the human aspect into energy efficiency legislation. Although the integration of the human component was still in its infancy at the time, it was seen to have immense potential for encouraging the adoption of greener behaviours at home. Despite the fact that most administrative measures currently in place are mostly focused on industrial energy use and infrequently on household energy consumption, it was noted that the compulsion of rules and regulations can easily result in a lack of social support [5]. Government policies can have a direct impact and moderate the effect of psychological factors on energy-saving behaviour [5]. The emphasis on the action’s dimension is another factor to consider in this subject. Traditionally, energy efficiency has been emphasised from an individual perspective by economists and behavioural scientists as an issue of personal choice. Nonetheless, the EU energy policy agenda still mainly ignores the social and collective dimensions of adopting practises towards domestic energy-saving [1].

This paper examines the demands and considerations that citizens take into account when making energy-related decisions, with a focus on measures to speed up this process. It is contextualised within the energy transition and people’s willingness to take direct action to mitigate and abate climate change. Specifically, the study presents a methodology to understand the behavioural factors that affect energy consumers’ decisions to implement energy-saving measures in the built environment. By learning more about these factors, we can better understand how to encourage people to shift towards more sustainable energy consumption models and increase social resilience in European cities. As of writing this paper, it is crucial to understand that human involvement is as important as technological solutions. There is a consensus that if individuals are not adequately driven to incorporate new technologies into their daily routines, even the greatest technology will fail [6]. To prevent potential issues in technology usage, it is better to identify and address fears and barriers as early as possible through qualitative and mixed methods.

Qualitative research is a valuable tool for exploring and gaining deeper insights into real-world issues. However, it can be challenging to implement and may not be recognized by some researchers or end-users [7], especially in the energy transition field. In this context, a proposed methodology involves conducting a qualitative study to create a novel taxonomy of the most relevant factors that affect households’ investment decisions [1], regarding the energy transition. The methodology aims to build a causal diagram [8] that identifies the reasons and factors that impact families and individuals when making energy investments related to any aspect of the energy transition. The transition refers to the global energy sector’s shift from fossil-based systems of energy production and consumption to renewable energy sources such as wind, solar, and lithium-ion batteries [9]. Causal models are built based on expert knowledge rather than existing data. To achieve this, we have defined a methodology, loosely based on the Delphi Method, to retrieve the knowledge of a panel of experts and build the causal diagram. This, in turn, has helped us design the taxonomy of the main factors affecting investment decisions. The transition from traditional fossil-based energy systems (such as oil, natural gas, and coal) to renewable sources (like wind, solar, and lithium-ion batteries) is a significant shift in the global energy sector. Experts use a methodology similar to the Delphi Method to build causal models based on their knowledge, rather than existing data. This approach helps retrieve the knowledge of a panel of experts and build a causal diagram, which is then used to design the taxonomy of the primary factors that affect investment decisions.

In this study, a taxonomy of factors that influence energy-related decision-making was obtained. A cross-sectional survey was then conducted to collect data on a specific population to determine the characteristics of the European population regarding behavioural factors related to energy transition. The study aimed to identify clusters of individuals with similar determinants that motivate their energy-related decisions, which could be targeted by policy measures [10]. The study examined the relationship between socio-demographic variables like gender, age, education level, and household income and energy consumption behaviour. The results show that there are no significant differences between males and females concerning direct energy curtailment [10].

This article brings up the factors that final energy consumers take into account when they make investment decisions related to the energy transition. The main outcome and contribution of this study is to highlight that these factors extend beyond financial considerations, which is different from the current focus on incentives. In essence, combining pecuniary rewards with responsible behaviour and knowledge can significantly influence energy-saving behaviour [11]. In addition, social commitment and support for policy measures ease the energy transition which main drive coming from changes in energy policy [12]. Regarding the challenge each region faces in transitioning to sustainable energy, emphasizing the need for tailored approaches, [13] this study provides a methodology that can help both propose to identify factors and prioritise them based on expert knowledge and to create common energy policies across regions [14].

The following sections provide a detailed explanation of our research. Firstly, the *Methodology* section presents the research methodology. Secondly, the *Results* section presents the findings of our research. The results begin by focusing on the construction of a 32-factor taxonomy obtained from expert knowledge. Then, they proceed to present the survey results. Next, the *Discussion* section interprets and analyses the survey results, along with findings from related projects in both European and Latin American contexts and limitations. Finally, the *Conclusion* section presents the conclusions and outlines objectives for future work, which aims to support the development of European energy-related archetypes and their intersection with a case study in four Latin American countries.

## Materials and methods

### Delphi methodology adaptation

This section outlines the methodology employed to identify the factors influencing household decision-making during the current energy transition. Due to COVID-19 restrictions in place at the time of this study, we adapted our methodology to an online framework, loosely following the traditional Delphi methodology. The Delphi method is favoured for its capacity to facilitate group decisions, often deemed more accurate than individual ones, making it a superior alternative to prediction markets or statistical groups. The primary objective of this iterative process is to guide a panel of experts towards consensus in the energy transition. Due to its simplicity and adaptability, the Delphi method has been applied in various contexts, including judgmental forecasting, project funding selection, and prediction of technology adoption.

The methodology is comprised of the following steps:

1. **Panel selection**: Assemble a group of authorities on one or more of the energy issues of interest, and diversity in demographics and geographical representation. Ensure that the experts have a broad range of perspectives and a deep understanding of the topic.
2. **Task assignment**: Assign specific forecasting tasks or challenges to each expert. These tasks should be well-defined and directly related to one of the energy aspects.
3. **Compile and summarize**: Collect all the initial forecasts and justifications and compile them. Summarize the information to provide clear feedback to the experts.
4. **Feedback and revision**: Share the compiled information and feedback with the experts. Encourage them to review and potentially revise their forecasts based on the feedback received.
5. **Final forecast**: The final forecast is created by aggregating input from all the experts. The final forecast reflects the collective wisdom of the panel.

**Fig 1:**
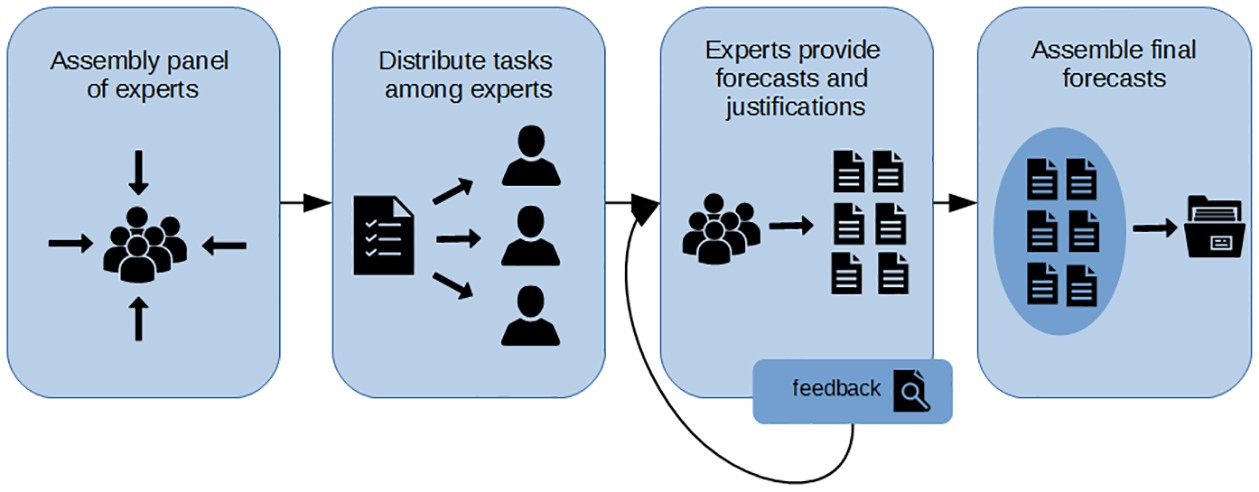
Visual representation of the Delphi method. Systematic and qualitative method of forecasting by collecting opinions from a group of experts through several rounds of questions.

After each round in the Delphi process, anonymous feedback and a summary of the experts’ responses are provided. This iterative process encourages refined thinking and may lead to more nuanced insights. In the final forecast (step 5), experts can cross-verify each other’s inputs; this will be done through workshops. Fig 1 provides a visual illustration of the aforementioned steps.

### Scenario-Based determinants analysis methodology

Delphi methods are most effective when dealing with rankings or quantitative objectives. However, in this particular task, the objective is to provide a qualitative description of reality, specifically the factors and determinants that make up the Causal Diagram. Therefore, an adaptation is necessary. To achieve this, the proposed methodology is as follows:

- Phase 0: **Literature review**. This phase helps identify the existing knowledge and gaps in the field, providing a foundation for further research.
- Phase 1: **Definition of the topics of interest**. In this study, four key aspects of the current energy transition were defined as the topics of interest: Buildings, Mobility, Flexibility, and Appliances.
- Phase 2: **Description of future scenarios**. We will create fictional scenarios for each of the four aspects of the energy transition, including Minimum, Probable, Plausible, and Ideal scenarios.
- Phase 3: **Expert panels and task assignments**. Separate panels of experts are established, each focusing on one of the energy aspects. Experts’ contributions are gathered through a series of designated tasks.
- Phase 4: **Creation of a common glossary**. coding and agreement of the answers collected in each aspect by several researchers.
- Phase 5: **Archetype crafting**. Creation of decision-maker archetypes at the household level.
- Phase 6: **Survey**. Conducting a cross-sectional survey to validate (or identify new) the defined archetypes.
- Phase 7: **Key factors and causal diagrams**. Selection of the most impactful determinants for each archetype and crafting of a Causal Diagram for them.

The recruitment period dates and the work sessions with experts are included in Table 1.

**Table 1:** Recruitment dates and working sessions with experts.

Linked phase	Activity	Start & End dates		Type of Session
		Europe	Latin America	
Phase 3	Recruitment	01/Jan/22—31/May/22	01/Jan/22—31/May/22	Online
	Individual contribution	01/Jul/22—20/Sep/22	29/Sep/22—13/Jul/23	Online
Phase 5	Workshop to sort determinants	20/10/2022 (Bilbao)	04/Nov/22 (México)	Hybrid (Bilbao)
			01/Nov/22 (Colombia)	
			02/Jan/23 (Chile)	In-person (Latin America)
			22/Jun/23 (El Salvador)	
Phase 6	Survey	15/Oct/22—15/Nov/22	11/Sep/22—31/01/2023	Online

Recruitment and working session dates with experts were scheduled in both the Europe and Latin America regions.

### Consent from

The informed consent statement confirms that all participants provided written informed consent to participate in the study.

#### Rights as stakeholder

- Your participation in this project is voluntary and no costs are derived for you. You have the right to withdraw at any point during the activity, for any reason, and without any prejudice.
- No personal data will be collected.
- All your contributions will be used only for the sole purpose of research (e.g. dissemination, outreach, etc.).
- Data contained in this survey will be kept at least until 5 years after the end of the project for auditing or reporting purposes by request of the funding agencies. A copy of your answers could be collected at the end of the survey following the steps provided by the system.
- The researchers in charge of this activity are Cruz E. Borges (cruz.borges@deusto.es) and Diego Casado-Mansilla (dcasado@deusto.es). You can contact them to solve any question related to this survey.

#### Consent form

By hitting the next button, you acknowledge that:

- Your participation in the action is voluntary.
- You are at least 18 years of age.
- You have read the background information that provides enough details about the project (purpose, expected duration and procedures of the study).
- You have been informed about your right to refuse to participate or to leave the activity at any moment without any justification.
- You have been notified of the contact persons, in the case you have questions or doubts during the activity.
- You have been informed how to get a copy of the consent form and your answers.
- You have had enough time to decide on your participation in the study.
- You have been informed about the questionnaire that you have been asked to complete.
- You have been informed about the storage procedures of the study data.
- You allow experts involved in the study under confidentiality agreements to utilize the information for the purpose of the study and only for this.
  * (Here, participants will find a checkbox) I agree to participate in the study.

#### Ethics statement

The Research Ethics Committee at the University of Deusto has qualified the project as FAVOURABLE (Reference: ETK-1/20-21). “the project is appropriate from an ethical point of view. According to the ethical standards and guidelines of Horizon 2020, the project purpose is clearly defined, and its implications regarding interaction with people and data gathering.

## Results

The presentation of results in this study follows a structured approach, beginning with scenario generation, where future energy transition scenarios are crafted. These scenarios undergo validation by a panel of experts to ensure their authenticity. Subsequently, an analysis of factors influencing energy-related investment decisions is conducted. The cross-sectorial survey collects data from diverse regions in Europe and Latin America, providing additional insights. Finally, the analysis of strata by socioeconomic variables allows us to grasp the role of socioeconomic factors in energy-related decision-making across strata. This methodical approach succinctly captures the intricate interactions within our study’s findings.

### Scenario generation

Inspired by Dunne et al. [15], this study uses speculative design as a suitable approach for identifying drivers of behaviour change or investment decision-making [15]. If experts are involved, these creative and speculative scenarios can yield inspiring and significant results overall, even though they represent a rather unconventional research methodology [15]. It’s true that a lot of intricate, socially based phenomena are difficult to measure or control through experimentation. It is recognised in consumer research that task-focused thinking is insufficient for creating and executing a successful system or framework. Hence, in order to provide outcomes that go beyond those that are “simply appealing,” it is crucial to understand users’ social drives and perceptions, including their expectations, identity, trust, and goals. In order to tackle this challenge, our study focuses on creating decision-maker archetypes. To achieve this, we have adopted a user-centric approach by consulting experts about the most significant determinants that impact the fictional scenarios created by the researchers. These scenarios are inspired by the ideas of Dunne et al. on speculative design and imaginary futures, as discussed in Anthony’s paper [15]. Dunne et al. were the pioneers of design speculation through fiction, and according to them, design speculation requires a bridge between the audience’s perception of their world and the fictional element of the concept. That was the reason why we proposed scenarios in which technology and information and communications technology IICT) advances (e.g., cutting-edge or emerging technology) were central to the proposals.

According to Xiao [16], speculative design centres on futures, which are seen as a range of possibilities that can be probable, plausible, possible, or impossible based on their likelihood of occurrence. A speculative design horizon of ten years is ideal for the near future. Founder of the Design Futures Initiative, Phil Balagtas, warns against excessive future projection, as it often leads to baseless speculation [17]. When predicting the future, we need to strike a balance between being too close to the present and too far into the future. Staying too close to the present requires thorough research to ensure accurate predictions, whereas being too far into the future can be purely speculative. Speculative design lies somewhere in between and helps us identify wild card scenarios, which are low-probability but high-impact events that can have a significant impact on society. This model highlights that the future is something we can shape and build by making the right choices today.

To create the causal diagram, we followed a “divide and conquer” approach by focusing on four use cases or application domains: appliances, building renovation, flexibility services, and mobility. This allowed us to obtain specific determinants for each use case, making the task more feasible. As part of our research, we have developed five hypothetical scenarios for each field. In these scenarios, each panellist must identify factors that influence their investment decisions. These scenarios are meant to represent different realities based on the concepts introduced in the literature review by Dunne, Rab [15], and Auger [18] for analysing various aspects.

- **Baseline**: These scenarios aim to depict a typical European city house in its current state, offering a general description of its various aspects.
- **Minimum**: In this context, the term “minimum” refers to the least amount of effort required to make progress toward the decarbonisation of specific areas of application, including appliances, building renovations, flexibility services, and mobility. Typically, these scenarios focus on changing behaviours instead of necessitating monetary actions.
- **Probable**: These scenarios project the most probable decarbonisation actions that citizens in any European city would take over the next few years.
- **Plausible**: These scenarios are less probable but not too unusual in some EU cities and households working towards decarbonisation goals.
- **Ideal**: It is highly unlikely that these ideal scenarios for decarbonisation will be achieved due to the significant social and cultural changes required.

A detailed description of the five types of scenarios for each energetic aspect can be found in the Project WHY Report [19].

### Scenario validation

In order to validate the scenarios, a group dynamic was carried out with an interdisciplinary and intersectoral panel of experts [20]. The list of experts and their affiliations can be found in Table 2. In this study, experts were presented with scenarios without pre-classification labels and were then required to read the scenarios and perform various tasks.

**Table 2:** Panel of experts.

Experts	Interdisciplinary	Intersectorial	International	Females
17	Technical, Economy, Societal, Public politics, Environmental research, Environment, Climate change	Academia, Energy & transport, Non-Governmental Organisations (NGOs), Third sector organisations (TSOs), Public administration, Environment & Climate, Policymakers	Spain, Germany, Hungary, The Netherlands, Belgium, Greece, Poland, Austria, and Italy	5

Expert panel for the dynamic of the scenario validation group.

**Fig 2:**
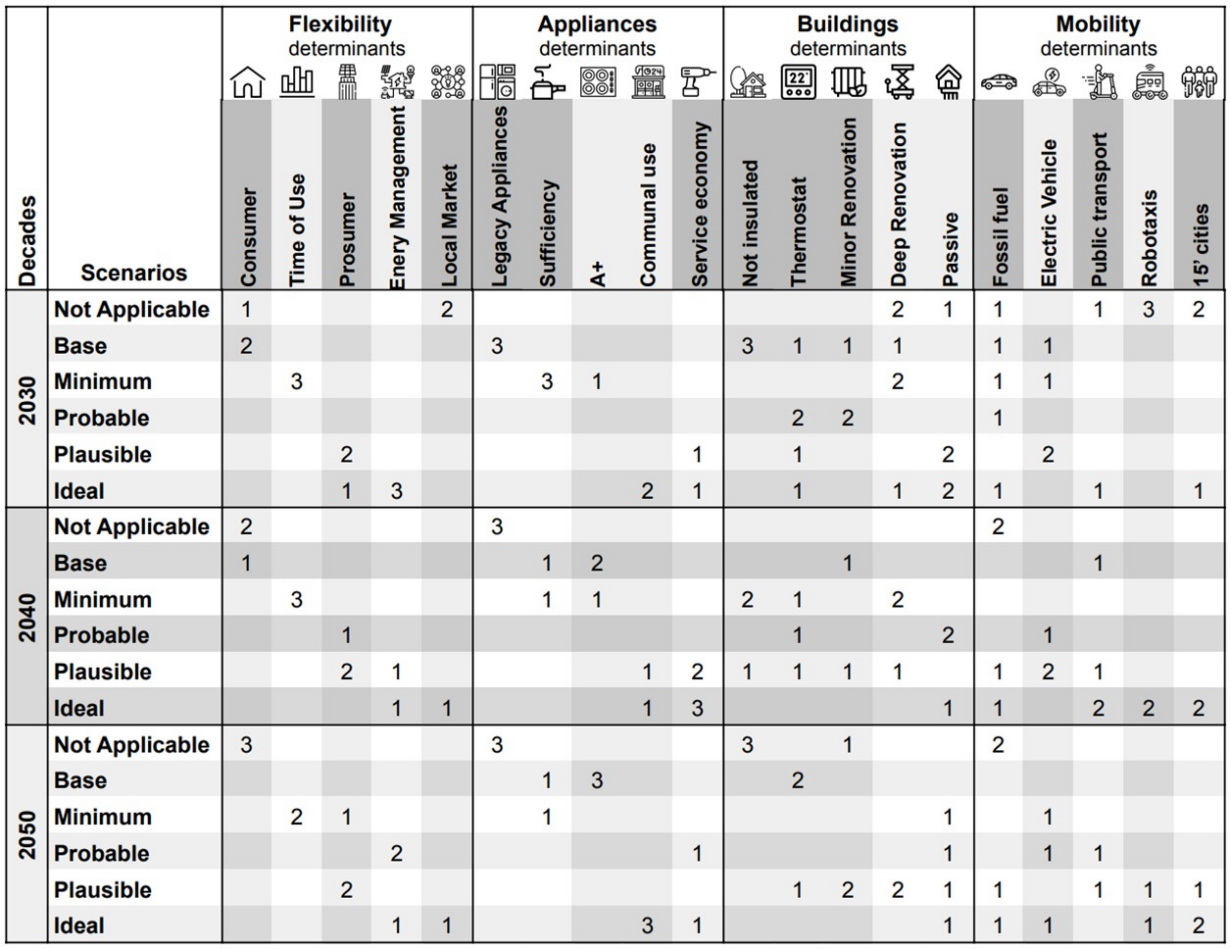
Mapping of scenarios to labels. Classification of scenarios by the experts into 5 categories for each energy aspect.

First, such scenarios had to be classified by the specialists as base, minimum, probable, plausible, and ideal (as introduced in the previous step). These classifications were made in the context of the period spanning the next three decades, from 2020 to 2050. The results of this evaluation can be seen in Fig 2.

During the experts’ analysis of the flexibility and appliance aspects, they positioned the scenarios near the diagonal and increased the scores every decade, which was expected. However, the trend was less clear for the building and mobility aspects. It is believed that since these two aspects were developed first, the task’s description may not have been entirely clear to the experts. In fact, one of the experts verbally expressed their concern during the session regarding this matter.

### Factors that affect investment decisions

After validating the scenarios, our goal is to create a comprehensive list of relevant factors that influence households’ decisions to vote or invest time and money to make these scenarios a reality. In order to do this, we brought together another panel of experts and requested that they list as many more factors as they could for each scenario. Inquiring about the experts’ individual reasons for reaching these conclusions as well as potential roadblocks to the above scenarios’ reality brought this phase to a close.

This section provides a summary of the panel’s organization, roles, and backgrounds. This was done to make sure that a variety of viewpoints and thoughts from different parts of Europe were gathered, as well as to address gender diversity in energy initiatives.

The project’s advisory board put together the panel of experts, which made an effort to include specialists from a range of disciplines, including technological, sociological, economic, psychological, and end-user. The primary features of the expert panel are enumerated in Table 3.

**Table 3:** Description of the expert panels.

Aspect	Number	Interdisciplinar	Intersectorial	International	Females
Building	7	All four fields	Academia, Industry & Public Authorities	Austria, Spain, Romania, Croatia and Poland	1
Appliances	13	All four fields	Academia & Industry	Austria, Norway, and Greece	3
Flexibility	7	All four fields	Academia, Industry & Civil Society	Spain, Austria, Germany, Sweden and Bulgaria	4
Mobility	7	Except psychology	Academia & Industry	Poland, Spain, Greece, Estonia, and Netherlands	2

Description of four expert panels, one for each energy aspect.

As can be seen, it was not always possible to achieve gender equity in the panels.

#### Individual work from the panel of experts

The entire activity was conducted online because of the COVID-19 pandemic restrictions. Experts were contacted via email, and following their consent to take part in the activity, a follow-up email was sent out with detailed guidelines (Task to be carried out [19]),

The task at hand was to provide compelling reasons for individuals to invest their time or money in each of the given scenarios. It is necessary to present barrier, extrinsic, and intrinsic elements in addition to any possible spillover effects.

All of the experts contributed more than a thousand distinct factors. Table 4 shows the contributions broken down by energy aspects and scenarios. There, it is evident that the distribution is somewhat homogeneous.

**Table 4:** Contributions by the experts.

	Minimum	Probable	Plausible	Ideal	Totals
Flexibility	84	70	72	65	**291**
Appliances	107	101	88	92	**388**
Buildings	58	97	67	70	**292**
Mobility	79	74	63	46	**262**
	**328**	**342**	**290**	**273**	**1233**

Expert contributions to the scenarios for each energy aspect.

#### Pair coding

In order to achieve this goal, each statement was first coded by a group of two researchers using the cognitive learning theory [21] Then, these two researchers met up in an effort to work out their mutual dejection. This led to a first codification, where most of the determinants were agreed upon; nonetheless, a third round of codification was conducted for the determinants that lacked agreement. Lastly, a third expert resolves any remaining potential disagreement by combining the responses from all four panels.

In the coding phase, 32 determinants were identified, based on research conducted by Hassenzahl [22]. The list of psychological needs that can lead to positive experiences, even when dealing with technical products or potential futures, was used as a reference to define the first-level category. This first-level category was defined by means of needs cards, which also served as a source of orientation and inspiration for the design of the interventions.

**Fig 3:**
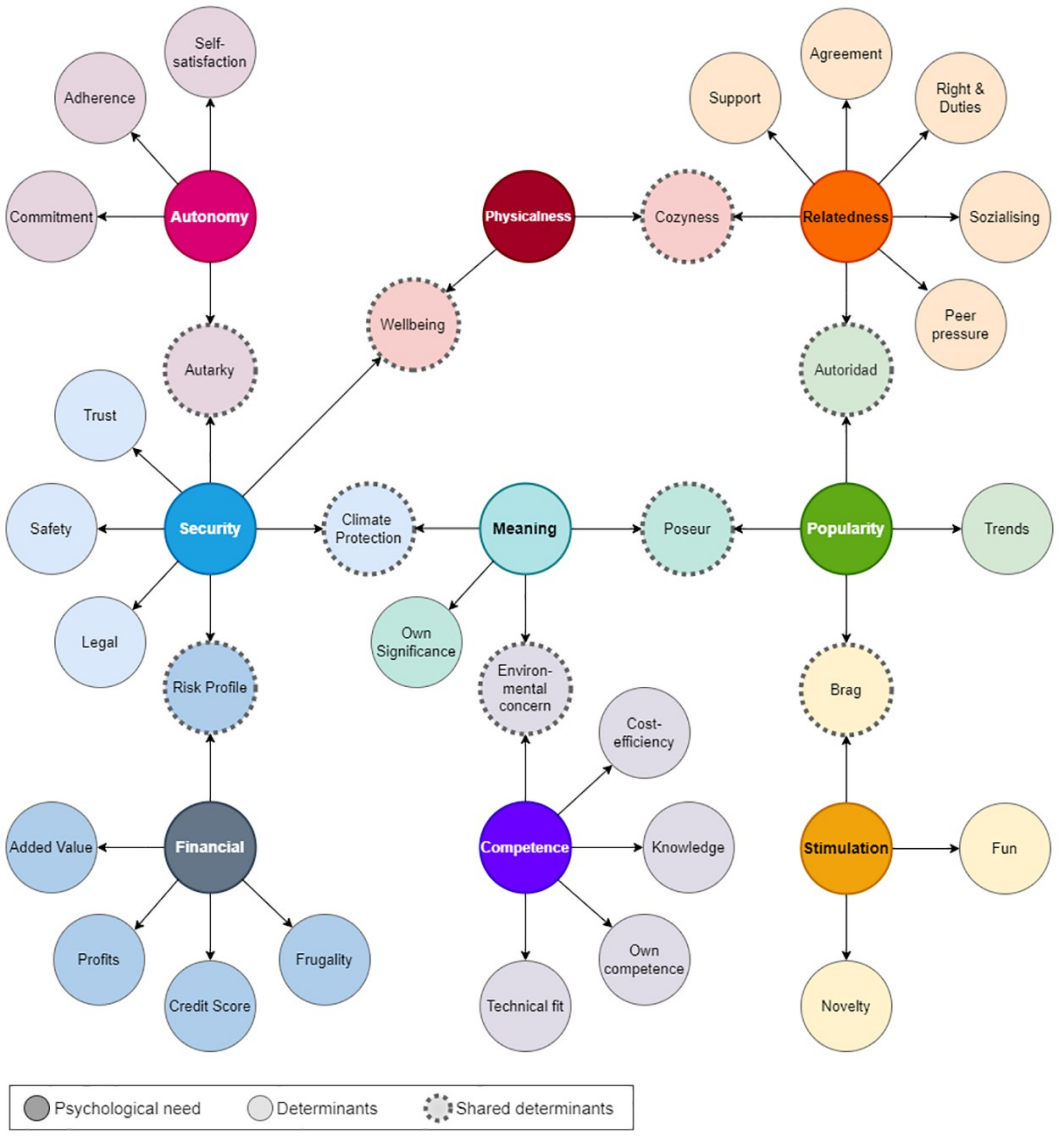
32-Factor taxonomy. The 32-factor taxonomy comprises two layers: the socio-physiological needs (in dark colour), and their associated/shared factors (in a light colour and dashed line).

Fig 3 shows a mind map of the distribution of these diagrams in a 32-factor taxonomy. Note that some determinants belong to the intersection of two categories in the social cognitive theory.

Table 5 displays the frequency of citation of each determinant in the different scenarios analyzed. Competence is the most frequently cited category in three of the four scenarios as well as in the average of all responses. In the data retrieved, financial needs are cited more often than other building topics. This is likely due to the higher cost of building infrastructure compared to other aspects. It is very important to emphasize that financial aspects are always present and are typically the second most important factor according to experts. In the third place, Relatedness refers to the influence that peers have on personal decisions. On the other hand, the Flexibility aspect is all about changing routines and shifting some loads to different times. Therefore, in line with appliances, Relatedness loads higher as it implies changes in the families’ behaviour. Despite being true for many aspects of life, it doesn’t seem to apply to mobility decisions. These choices are usually more individualistic with a lower relevance placed on social aspects. In this context, security appears to be the most important factor, as people prioritize personal safety and trust when making changes. Physicalness and stimulation are less important factors and are found at the lower end of the determinants’ hierarchy. This suggests that investments made for fun or to improve comfort and well-being at home are not as important as other factors. While there are some intersections between security and physicalness, the results are somewhat unclear.

**Table 5:** Distribution of determinants.

Needs	Flexibility	Appliances	Building	Mobility	Average
Financial	18%	13%	**32**%	16%	**19%**
Security	10%	11%	10%	20%	**13%**
Competence	**26**%	**29**%	15%	**22**%	**24%**
Autonomy	7%	6%	4%	5%	**5%**
Physicalness	0%	1%	5%	4%	**2%**
Relatedness	24%	21%	18%	15%	**19%**
Stimulation	3%	4%	3%	3%	**3%**
Popularity	7%	10%	8%	7%	**8%**
Meaning	5%	6%	6%	7%	**6%**

Weight distribution across psychological needs in energy dimensions.

### Cross-sectorial survey

A cross-sectional survey offers momentary insights into a particular group. In this instance, our objective is to identify groups of people who have similar factors impacting their choices on the energy transition and to define the European population using a 32-factor taxonomy (explained in List of determinants [19]). Identifying clusters allows validating investment archetypes and comparing them to initial identifications. Which are the key elements influencing investment decisions in the energy transition, according to the survey [23] seeks to evaluate the determinants (factors) that cause consumers to engage in the energy transition in four distinct contexts or scenarios: Flexibility markets (e.g. PV panels), Energy efficiency (e.g. building insulation), Mobility (e.g. electric vehicles), Energy conservation (e.g. sharing economy). The gathered raw data and variable descriptions can be found online at https://doi.org/10.5281/zenodo.7382924.

**Fig 4:**
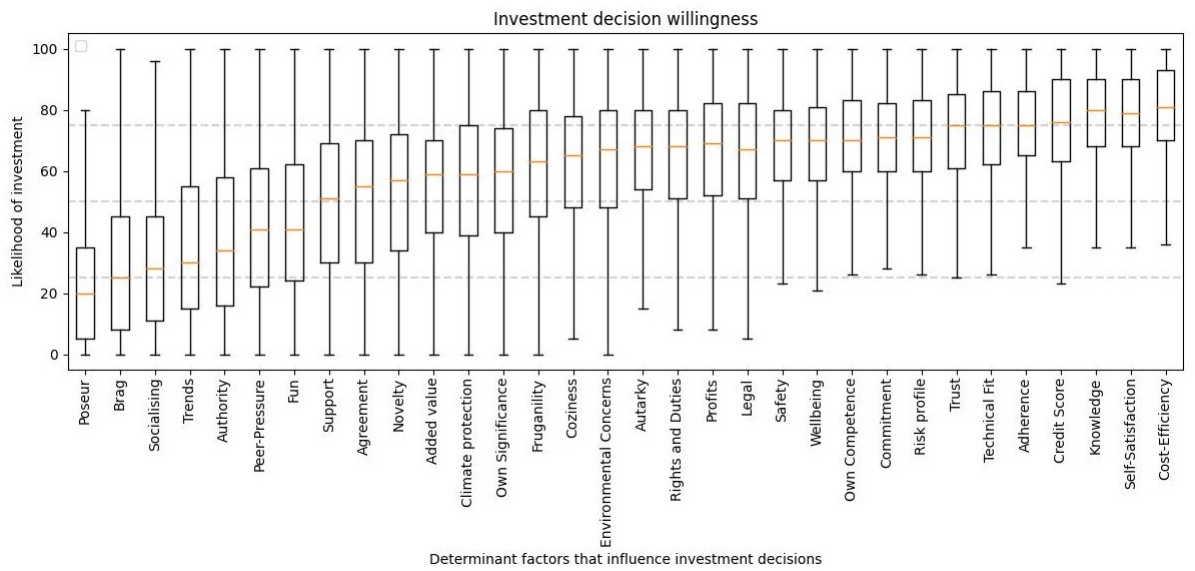
Investment decision willingness. Investment decision willingness, sorted factors from left to right by people’s priority.

When it comes to investment decisions, Fig 4 shows the relative importance of various factors. These factors are rated on a scale of 0 to 100. Generally, the most significant factors have a mean value close to or above the 75th percentile, while the less impactful factors have a mean value close to or below the 25th percentile.

### Analysis of strata according to socioeconomic variables

A non-parametric statistical hypothesis test of similarity (Wilcoxon signed-rank test) has been carried out. Significant differences were found between the answers from Europe and Latin America (p-value = 0.0410) and between the group of persons with varying educational levels (p-value = 0.0033). Nevertheless, Gender, Age, or Savings Capacity does not significantly impact interest levels in investments related to appliances, heating, lighting, electro-mobility, or related activities. The differences observed for the country of residence, are spread in several categories suggesting these differences are systemic while the ones related to the level of education are more concentrated on the Autonomy and Competence aspects which make sense.

Finally, we examine the differences in terms of the distribution of the determinants by different socioeconomic strata. Table 6 presents the distribution of the determinants in each stratum. Three levels of interest/concern can be discerned from the socioeconomic characteristics in the survey: low (-, yellow), medium (=, grey), and high (+, blue). These levels are determined based on a scale ranging from 0 to 100, divided into three bands: 0-33 for low, 34-65 for medium, and 66-100 for high interest/concern.

**Table 6:** Differences among groups based on socioeconomic variables*.

Needs	Factors	Region		Edu.Lev		Savings		Age		Gender		Average level
		EU	Latam	Pre-Uni	University	Less than 10%	More than 10%	18-55	Over 55	Male	Female	
Financial	**Profits**	**=**	+	+	**=**	+	=	+	+	+	+	+
	**Credit Score**	+	+	+	**=**	+	+	+	+	+	+	+
	Risk Profile	+	+	=	=	+	+	+	+	+	+	+
	Added Value	=	=	=	=	=	=	=	=	=	=	=
	Frugality	=	=	=	=	=	=	=	=	=	=	=
Security	Legal	=	=	=	=	+	+	+	+	+	+	+
	Trust	+	+	=	=	+	+	+	+	+	+	+
	Safety	+	+	=	=	+	+	+	+	+	+	+
	Climate Protection	+	+	=	=	=	+	=	=	=	=	=
Competence	Cost-Efficiency	+	+	+	+	+	+	+	+	+	+	+
	Knowledge	+	+	+	+	+	+	+	+	+	+	+
	**Own Competence**	+	+	+	**=**	+	+	+	+	+	+	+
	Technical Fit	+	+	=	=	+	+	+	+	+	+	+
	**Env. Concerns**	+	**=**	+	**=**	+	+	=	+	=	+	+
Autonomy	Self-Satisfaction	+	+	+	+	+	+	+	+	+	+	+
	**Commitment**	+	+	+	**=**	+	+	+	+	+	+	+
	**Adherence**	+	+	+	**=**	+	+	+	+	+	+	+
	**Autarky**	**=**	+	=	=	+	=	+	+	+	=	+
Phy	Wellbeing	+	+	=	=	+	=	+	+	+	+	+
	**Coziness**	**=**	+	=	=	=	=	=	=	=	=	=
Relatedness	**Rights and Duties**	**=**	+	=	=	+	+	+	+	+	=	+
	Peer-Pressure	=	=	=	=	=	-	=	=	=	=	=
	Support	=	=	=	=	=	=	=	=	=	=	=
	Socialising	-	-	=	=	-	-	-	-	-	-	-
	Agreement	=	=	=	=	=	=	=	=	=	=	=
Stimul	Novelty	=	=	=	=	=	=	=	=	=	=	=
	Fun	=	=	=	=	=	-	=	=	=	=	=
	Brag	-	-	=	=	-	-	-	-	-	-	-
Popu	**Trends**	-	**=**	=	=	-	-	-	-	-	-	-
	**Authority**	-	**=**	=	=	=	-	-	=	-	-	=
Mean	Own Significance	-	-	=	=	=	=	=	=	=	=	=
	**Poseur**	**=**	+	=	=	-	-	-	-	-	-	-
Significant diff.		**8**		**6**		7		2		3		
(Wilcoxon) P-Value:		**0.0410**		**0.0033**		0.3458		0.4237		0.0726		
*It is recommended to view the figure in colour for a more comprehensive understanding												
+	High-level of interest											
=	Medium-level of interest											
-	Low-level of interest											
Note: Darker colours mean a Significant difference												

Significant differences were identified between groups categorised by their socioeconomic strata.

Wilcox analysis was used to investigate possible significant differences between two categories of groups according to socio-economic variables, such as (1) region (Europe vs. Latin America), (2) savings (percentage of income saved for energy investment), (3) gender identity (male/female), (4) age (two main age ranges) and (5) educational level (pre-university or university and higher education). The results, together with the calculated p-values, are presented in Table 7.

**Table 7:** Analysis of sister projects.

NEEDS:		Financial		Competence					Relat.		Security			
Num	Project	Profits	Frugability	Cost-Efficiency	Knowledge	Technical Fit	Env. Concerns	Own Competence	Socialising	Agreements	Climate Protection	Legal	Wellbeing	TOTAL
1	REFEREE	✓				✓	✓						✓	4
2	REScoopVPP	✓				✓	✓		✓	✓			✓	6
3	frESCO	✓				✓	✓		✓				✓	5
4	sEEnergies	✓		✓		✓	✓				✓			5
5	MICAT	✓		✓	✓						✓			4
6	ENEFIRST	✓	✓	✓	✓	✓								5
7	SENTINEL			✓		✓	✓				✓			4
8	DECIDE				✓							✓		2
9	EC^2^				✓		✓					✓		3
10	NEWTRENDS				✓	✓	✓				✓			4
11	NUDGE			✓	✓	✓	✓							4
12	GreenSoul			✓	✓	✓	✓							4
13	ENCHANT			✓	✓	✓								3
14	EVIDENT				✓	✓	✓							3
15	EU 1.5° Lifestyles				✓		✓							2
16	ENPOR					✓	✓							2
17	SocialRES						✓		✓					2
18	RES4CITY				✓	✓	✓	✓					✓	5
19	ebalance-plus				✓	✓	✓	✓		✓		✓	✓	7
Number of Projects:		6	1	7	12	14	15	2	3	2	4	3	5	
Proportion of projects by need:		32%		79%					16%		26%			

Comparative analysis of sister projects highlighting the factors and needs they address, in contrast to those addressed by our research.

## Discussion

This section reveals valuable insights into the complex interplay of behavioural factors in the context of energy efficiency, sustainability, the transition to a low-carbon society, and resilient societies in cities [24]. In this discussion, we summarize the key findings from these projects and offer interpretations and implications, acknowledging limitations, along with the findings from an analysis of several sister projects. The results obtained by the introduced methodology allow the building of a 32-factor taxonomy [19] that influences people’s decisions and get insights into the varying degrees of influence regarding actions related to energy transition.

Based on the modelling energy efficiency and energy demand project review (Table 7), The energy transition takes place in two main sectors: demand-side and supply-side sectors; most of the projects are focused on households and energy communities sectors from the demand-side [25, 26], this also addresses the need to cover the lack of accuracy in the energy consumption to the household level and taking into account the factors strongly linked to the personality of the energy consumer, the kind of archetypes.

The methodology introduced in this research [19] was designed to extract human expert knowledge to identify the factors influencing people’s decisions regarding actions related to energy transition (Table 5). Its capability to provide valuable insights and guidance for policymakers, researchers, and stakeholders lies in several key aspects: Informed Behavioural Insights, Decision-Making, Tailored Interventions, Measurable Outcomes, Adaptability, and Public Engagement.

The findings presented in Table 5 provide insights into the varying degrees of influence that behavioural factors have within the energy aspects, highlighting the nuanced interplay of these factors in decision-making processes. According to the experts (Table 5), in three out of the four energy aspects, the factors associated with Competence take precedence. Following closely in terms of importance are the factors linked to Relatedness and Security. However, in the context of the Buildings aspect, financial factors emerge as the most significant, while in the other three energy aspects, they assume a third-place ranking. This suggests that end consumers tailor their decision-making processes based on the specific energy aspect they are dealing with.

According to experts, Competence factors and Relatedness are the most significant factors, surpassing financial (Table 6), and the survey results confirm this view (Table 5). Although experts consider Relatedness as the second-order priority, survey respondents prioritize Autonomy and Security as second and third, respectively, ahead of financial needs. Both experts and respondents agree that there are more important needs than financial ones.

Several projects collectively shed light on the role of various behavioural factors in the decision-making process, influencing individual and collective actions, decisions, and policies. These projects are presented in Table 7.

Table 7 displays the factors considered in the projects and their links to different needs. The most common focus is on competence factors, at 79%, followed by financial factors (32%), security (26%), and community-related factors (26%). Unlike the distribution in Table 5, competence factors are prioritised over financial factors, security, and relatedness. These results are consistent result in with Table 5. However, it’s crucial to acknowledge that intervention success improves when factors addressing multiple needs are considered based on individual priorities. For example, competence factors are prioritised in energy-related areas like flexibility, household appliances, and mobility but are less essential in building-related contexts.

Based on Table 7, common behavioural factors are addressed indicating their significance in the context of decision-making processes and getting an understanding of which factors have been their primary focus, which leads to the following conclusion:

- **Financial factors**. The projects REFEREE [27, 28], REScoopVPP [29, 30], frESCO [29, 31], sEEnergies [32, 33], MICAT [34–36], and ENEFIRST [37] prioritize economic gains (profits) and losses associated with energy efficiency measures, indicating a strong focus on financial factors. Additionally, the ENEFIRST project incorporates Frugality to improve energy efficiency, achieve cost savings, and reduce energy consumption. ENEFIRST project mentions price as a key trigger of consumer behaviour, indicating a focus on pricing strategies and their influence on consumer choices. REFEREE is quantifying the benefits of energy efficiency may involve considering employment opportunities, suggesting an interest in economic and employment-related factors. The financial aspects of providing energy efficiency and demand flexibility services are highlighted by frESCO, suggesting a focus on economic consideration.
  In contrast, DECIDE [38] mentions that many households are not able to afford to start or be part of an energy community, indicating an economic dimension related to *affordability*; *Energy poverty* is mentioned by ENPOR [39, 40] in the context of increasing energy costs, highlighting economic factors and the financial challenges faced by households.
  This study demonstrates that financial factors are not the sole drivers of behavioural change across all energy transition contexts. Instead, decision-makers in households prioritize other factors as more crucial, especially flexibility, appliances, and building contexts.
- **Competence factors**. Regarding *competence factors*, all projects consider one to four factors, These projects share a common emphasis on *Environmental concerns* (15 projects), particularly related to environmental sustainability, including carbon emissions reduction and energy conservation. Additionally, they prioritize *Technical fit* (14 projects) considerations, such as technology deployment, compatibility, and integration; in addition, the ebalance project [41] is focused on the development of hardware and software elements, grid integration, and flexibility mechanisms. In 12 projects, *Knowledge* acquisition, research, and data analysis are consistently highlighted as essential for understanding and promoting energy behaviour change; and REST4CITY [42] emphasizes the need to up-skill academics and professionals in sustainable technologies. Moreover, these projects address *Cost Efficiency* (7 projects) through cost-effectiveness assessments and transparent cost-benefit analyses. EC^2^ emphasizes the need for empowered energy citizens and energy communities, indicating the significance of social factors in shaping the energy transition. In addition, GreenSoul [43], ENCHANT [44, 45], EVIDENT [46–48], and EU 1.5° Lifestyles [49, 50] projects, introduce a strong emphasis on behavioural science principles. GreenSoul aims to change energy consumption behaviour, while ENCHANT tests behaviour-focused interventions. EVIDENT examines behavioural biases affecting energy savings, and EU 1.5° Lifestyles relies on *behavioural science* to induce low-carbon lifestyles.
  The results of this study affirm the significance of competence factors, which are deemed more crucial than financial factors in decision-making. Prioritizing these factors in the formulation of energy policies holds greater importance, particularly when considering changes in energy policies related to *environmental concerns* and responsibilities.
- **Relatedness factors**. REScoopVPP, frESCO, SocialRES, and REST4CITY [42, 51–53] projects emphasize both social factors related to consumer behaviour, social connections, and cooperation among individuals and organizations; and second, the well-being of communities in the context of energy efficiency and renewable energy.
  In this study, besides the “socializing” factor (feeling connected to others and part of a community), brings focus on the obligations and benefits in a community (rights and duties), the influence of peers enforcing social norms (peer pressure), contribution to community improvement and social causes (support), and the understanding and cooperation among peers (agreement) to enhance the social acceptability of renewable energy strategies and energy democracy.
- **Policy instruments**. The reviewed projects collectively aim to engage with and influence various policy instruments in the context of energy efficiency, sustainability, and the transition to renewable energy sources. They seek to provide policymakers with tools, insights, and strategies to estimate the impact of policy actions, assess the effectiveness of policy instruments, and promote sustainable behaviours. Their interests range from regulatory aspects related to renewable energy deployment (REScoopVPP, frESCO, NEWTRENDS [54]) and energy efficiency policies (sEEnergies, MICAT) to the broader transition to a low-carbon energy system (SENTINEL [55]). Additionally, some projects focus on understanding and addressing barriers and facilitators to energy citizenship and community empowerment (EC^2^ [56, 57]) and promoting energy efficiency through policy-driven behaviour change (NUDGE [58], ENCHANT, EVIDENT). Others target specific aspects like energy poverty (ENPOR) and social innovation in the renewable energy market (SocialRES), reflecting their commitment to shaping policy instruments for inclusive and sustainable energy solutions.
  The results underscore that, on one hand, key strategies for reducing energy consumption include the promotion of energy-efficient technologies and behavioural changes, alongside policy interventions [59]; Examples of these interventions are energy labelling [60, 61], incentives for energy-efficient renovations [59, 62], and the promotion of renewable energy sources. On the other hand, the success of these policies, particularly in encouraging consumers to adopt energy-efficient habits, critically depends on the effectiveness of behavioural change. A deeper socioeconomic understanding (Table 7) of climate change is pivotal for targeted policy planning and implementation [63].

The projects reviewed (Table 7) refer to these needs as motivating or hindering factors influencing end-consumers’ decision-making in support of the energy transition. This study underscores that factors related to competence, relatedness, and security hold significantly more sway over people than purely financial factors, a viewpoint corroborated by the survey results (Table 6). Therefore, this study emphasizes that interventions should go beyond addressing only financial needs. A deeper comprehension of people’s requirements can inform the development of policies aimed at fostering changes in residents’ heating system usage, thereby reducing their carbon footprint.

This is fully aligned with the results found in the current research as it seems that internal factors such as beliefs, values, or environmental concerns are the prominent drivers that foster a change in people’s daily behaviour to invest time and effort in reducing carbon and energy footprint in their dwelling. Besides, the dominant external factors for such pro-environmental behaviour to occur seem to be peer pressure, social comparison, and social norms. Being these ideas aligned with the expected results, these intrinsic motivations (i.e., beliefs, values, or environmental concerns) arise as the main dwellers for energy-related choices. Nevertheless, depending on the diversity of the individual, the weight given to these intrinsic motivations may vary for each person. Thus, at this point, the discussion can be about whether the external factors could be a differential key point to drive the behaviour complementary to the intrinsic determinants. Apart from these explained above, other specific determinants are mentioned less frequently by the experts. The main determinants discussed appear as the main concepts, and the other determinants appear in each specific scenario after the main topics have already been covered.

The second idea that should be discussed is that the main group of determinants is related to competence. This finding should be highly taken into account since it appears in all the scenarios in the first or second place (Table 5). Thus, although the main determinant for sustainable behaviour can be easily identified by taking into account these results, it is still not clear how to boost that competence feeling in people. Relatedness and financial factors are tied in second place, being also in concordance with the main intrinsic motivations exposed by Deci et al [64]. Relatedness is linked to people and family. This is in line with human beings, who are social entities that live in a community. The concept of financial is related to security, and therefore it is also linked to people’s basic emotional needs.

Finally, it seems that flexibility and appliances follow a quite similar distribution. This may be due to the assimilation that a flexibility provider is just “another appliance” or due to the undiscovered factors. Moreover, security determinants seem to be very relevant for mobility. This may be related to the ability to secure access to the workplaces and other daily chores and maybe a key determinant when analysing and reflecting on future work related to this specific scenario. Besides, financial determinants seem to be very relevant for buildings. In this regard, the high investment cost related to any action in this regard is probably the main cause of this result.

Concerning the factors impacting individuals’ investment choices within the presented scenarios illustrates their relative importance, rated on a scale from 0 to 100. The most significant factors typically exhibit a mean value near or exceeding the 75th percentile, while the less impactful factors tend to have a mean close to or below the 25th percentile, as is shown in Table 4. The last column (average level) in Table 7 presents the average score of each determinant of the population. What can be seen is that determinants related to economic (financial and security) and individual factors (competence, autonomy, and physicalness) are positively scored by the population while those related to the community (Relatedness, Stimulation, Popularity and Meaning) are negatively scored. This is just another reflection of the societal trend towards more individualistic behaviour [65] that will have a profound impact on the development of energy communities as these are linked to collective decision-making [66]. This should be further investigated as a large part of the European strategic goals for de-carbonising the economy lies on the shoulders of energy communities [67].

### Limitations

This study’s methodology primarily involves the aggregation of human expert knowledge from professionals specialising in the field of energy transition. It is important to note that some data were collected during or near the COVID-19 pandemic, which could introduce a bias in the results, which predominantly represent the viewpoints of these experts.

## Conclusions

More to the point, in this paper we have presented a methodology to build a taxonomy of determinants that affect decision-making in households related to investment in the energy transition. 32 determinants were found by a panel of experts closely related to the theory of planned behaviour. Distribution of these determinants in Europe.

The next steps include the clusterization of the population concerning the determinants in order to build archetypes [68]. To address this complexity, a set of seven archetypes of behaviours is built using the taxonomy. These archetypes consist of a set of determinants sorted following the Transtheoretical Model (TTM), making a causal diagram. Unfortunately, neither the seven archetypes nor their causal diagrams are validated, so proper validation has to be carried out.

The validation will consist of three main activities: (1) a cross-sectional survey to validate (or create new ones) the defined archetypes; (2) a longitudinal survey to sort determinants of the archetypes found in the stages of the TTM; and (3) expert workshops to sort determinants of the archetypes found in the stages of the TTM. The cross-sectorial survey has been carried out.

We contribute not only with a behavioural 32-factor taxonomy, but this study also aims to illustrate the causal relationships between these factors. By representing different types of energy end-users as archetypes or persons, which stand for behavioural patterns in the decision-making process, this study implements causal modelling of these factors. The goal is to give policymakers a tool to explore the impact of interventions on these causal paths and predict how people will react to changes in energy policy.

The results of the p-value analysis from the survey highlight the significance of differences between groups defined by socio-economic variables. Specifically, in terms of gender, there is a notable difference [69] in the prioritization of 9 out of 32 factors. Additionally, the prioritization of factors in Europe differs significantly from their prioritization in Latin America.

The results of the p-value analysis from the survey highlight the significance of differences between groups defined by socio-economic variables. There is no significant difference in gender, age, and savings; Table 6 shows a difference in the prioritization of 12 out of 32 factors. Similarly, Table 6 also shows that the prioritisation of factors in Europe differs significantly from that of Latin America.

Numerous projects emphasize the role of human behaviour in energy efficiency, often focusing on factors addressing a single need or multiple needs but limited to one or two factors (Table 7). This study, however, presents nine needs and 32 factors that experts believe energy policy should target to enhance social commitment to energy efficiency.

From this stage, the next will focus on the causal modelling of human behaviour within European archetypes (types of individuals). It involves a thorough analysis of the causal pathways in the decision-making processes of European archetypes and identifies the behavioural factors that energy policies can intervene in to promote energy-saving behaviours. Additionally, we plan to apply the proposed methodology to construct archetypes within the Latin American context, serving as a validation step. This will help us understand how Latin Americans behave in the context of the energy transition and identify differences between the two contexts, providing valuable insights for further research.
